# Numerical Tensile Damage Procedure Analysis of Angle-Ply Laminate Using Multi-Scale RVEs with Smear Crack Models

**DOI:** 10.3390/ma15062002

**Published:** 2022-03-08

**Authors:** Qianwen Wang, Fa Zhang, Zhenqian Lu, Dongfeng Cao, Xiwen Jia

**Affiliations:** 1 Jiangsu Province Engineering Research Center of Biomass Functional Textile Fiber Development and Application, Yancheng Polytechnic College, Yancheng 224005, China; ycfywqw@126.com; 2 Beijing Key Laboratory of Civil Aircraft Structures and Composite Materials, Beijing Aeronautical Science & Technology Research Institute of COMAC, Beijing 102211, China; zhangfa@comac.cc; 3 School of Textile and Garment, Yancheng Institute of Technology, Yancheng 224051, China; luzhenqian2003@126.com; 4 State Key Laboratory of Materials Synthesis and Processing, Wuhan University of Technology, Wuhan 430070, China; cao_dongf@whut.edu.cn

**Keywords:** multi-scale, smear crack, composite, damage, UMAT

## Abstract

This paper reported the tensile failure strengths and damage procedure of composite laminate manufactured from the toughened-epoxy T800 prepreg at multi-scale levels. According to the exterior and interior distinction of each layer in laminate, the macro/mesoscale representative volume element (macro-RVE, meso-RVE) was first constructed, respectively. Then the micro-scale representative volume element (micro-RVE) with a hexagonal fiber-packed pattern in the interior zone of each layer in the laminate was finally determined on the principle of the same fiber volume fraction between the composite laminate and multi-scale RVEs. In the multi-RVEs analysis, the mechanical failure strengths of each scale model were transmitted from the last-scale model’s homogenization, such as the meso-RVE from micro-RVE and the macro-RVE from meso-RVE. Based on our previous report, the innovative multi-scale damage and post-damage models on the concept of the smear crack were improved fully and incorporated by user-defined material subroutines (UMATs), such as in the addition of multiple cracks co-coupled, which makes it predict the element damage procedure. The averaged mechanical responses with damage mechanism of multi-scale RVEs under tensile, compressive, or shear loadings were obtained wholly by the homogenization method. The macroscale tensile damage initiation and propagation procedure were analyzed in detail including their global/local responses, being extended to comparison with experimental results.

## 1. Introduction

Due to the characteristics of inherent anisotropy and heterogeneity, the damage mechanisms of composites are complex occurring at multi-scale levels, such as matrix cracking, fiber breakage, delamination and debonding, etc., [1] which make efficient and accurate numerical predictions play an increasingly important role in their design and application in various industries [2]. In order to clarify the inherent progressive failure processes of laminated composites from a fiber-matrix level, lamina level, laminate, and component level, a multi-scale damage analysis incorporating the physics of the above-mentioned failure may provide an accurate prediction of failure strength and mechanisms [3,4,5]. Recently, the use of the micro-meso-macro concept in multiscale analysis is widely performed by transferring mechanical parameters and damage details from one scale to the next level.

To predict this challenging response resulting from the complex multiscale nature of failure, an improved understanding of the progressive damage evolution in laminated composites has been reported numerically. At a micro-scale level (e.g., fiber/matrix), to reveal the initiation and evolution procedure of damaged zone in detail, the discrete fiber-level micromechanical model is a preferred approach to characterize transverse crack formation and longitudinal fiber failure [5,6,7,8,9,10,11,12,13]. At the mesoscale level (e.g., laminar or ply), to present the experimentally-observed delamination migration phenomena, the initiation and propagation, such as the delamination and matrix cracks were modeled within the Floating Node Method (FNM) elements [14]. The matrix yielding was found to play a crucial role in this process, which leads to the fibers first failing in the compressive side in the numerical and analytical models for the formation of kink-band [15,16]. Moreover, regarding the out-of-plane strength of the curved composite laminates, matrix failure and delamination are the two dominant damage modes [17,18,19]. For the macroscale analysis (e.g., laminate with different angle-ply layers composite), the averaged stress–strain responses obtained at the micro-level are incorporated to reveal the macroscale constitutive behavior and the damage mechanism [5,20,21]. From the constituent properties measured from lamina-level coupon tests, A two-scale, micromechanics-based model was utilized to predict the progressive damage and failure responses of composite laminate [22,23,24,25] and the initial transverse micro-cracks [26]. The outstanding advantage of multi-scale models is their ability to capture the damage initiation and evolution at multiple length scales revealing their damage physics, obviously.

Using the principle of the real layer distribution in laminate and consistent fiber volume, a mesoscale representative volume element (meso-RVE) for the ‘cured prepreg’ layer and a micro-scale repeating volume element (micro-RVE) was constructed. Based on our previous works, as stated in the UMAT subroutine only with a single-crack (e.g., a normal crack, shear crack) mode on the concept of the smear crack [27,28], the damage mechanism of the mesoscale analysis in layer model and the macroscale analysis in the laminate model were introduced with both double-crack mode (e.g., normal-shear coupled crack) and triple-crack mode (e.g., normal-shear-normal coupled crack). All possible failure modes (e.g., single crack mode with six types, double crack mode with 18 types and triple crack mode with 18 types in detail as stated in chapter 3.2 with the principle of the smear crack concept were incorporated in the current multi-scale user-defined subroutines (UMATs). In the further loading process, the above-mentioned innovative strategy brings the possibility to feature the damage procedure of laminate composite numerically, such as matrix cracking, fiber fracture/splitting and delamination, etc., which behaved distinctive to the numerical response of the delamination based on the cohesive zone model (CZM) [29,30,31] or the continual damage model (CDM) [32,33,34,35]. The incremental stress–strain relations were applied in UMATs to avoid the convergence problem after the crack occurred.

In contrast to physical cracked modes of the microstructure and crack modes of the fiber reinforced composite plates, corresponding to virtual cracked modes [36,37], this paper strives to investigate the damage procedure for different failure modes with the smear crack approach. In the current multi-scale analysis, the mechanical engineering parameters with failure strengths of each scale model were predicted, homogenized and transferred from the current scale level to the next one, such as the meso-RVE from micro-RUC and macro-RVE from meso-RVE. The damage and post-damage constitutive responses at multi-scale FE models were conducted successfully with UMATs incorporated by ABAQUS/Standard.

## 2. Experimental System

**Figure 1:**
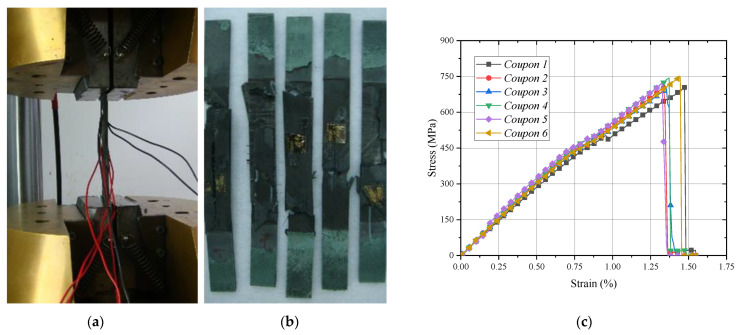
Tensile experimental system and damage morphology of angle-ply laminate specimens: (**a**) Test system; (**b**) Fracture morphology; (**c**) Stress–strain cure.

**Figure 2:**
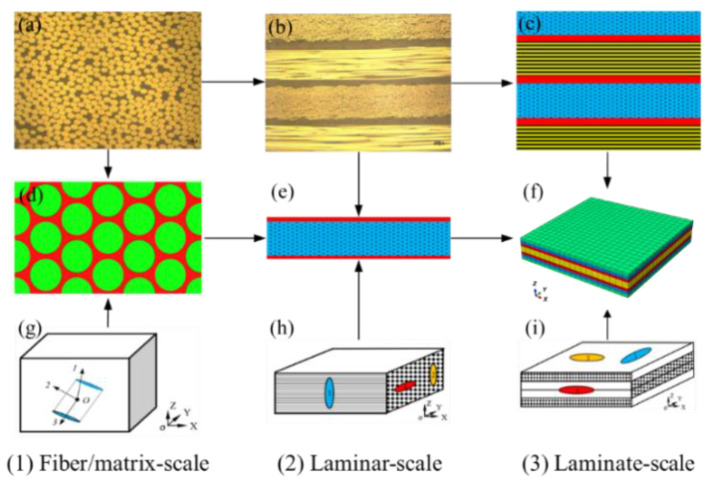
Multi-scale RVEs with damage models of angle-ply laminate: (**a**) Fiber-packed patterns in layer; (**b**) Layer-packed in laminate; (**c**) Macro-scale FE model; (**d**) Micro-scale FE model;(**e**) Meso-scale FE model; (**f**) Macro-scale RVE;(**g**) micro-scale smear crack model; (**h**) Meso-scale smear crack model; (**i**) Macro-scale smear crack model.

The experimental specimens were manufactured from the T800 carbon prepreg angle-ply laminate with [+45/−45/0/90]_s_. The T800 prepreg consists of T800 carbon fiber (supplied from Toho Tenax Co., Ltd., Tokyo, Japan) and toughened-epoxy (supplied from Cytec Industries Co., Ltd., Woodland Park, NJ, USA) with a normal thickness of 0.185 mm and a fiber volume fraction of 57.3%; the mechanical parameters of fiber and resin in T800 carbon fiber prepreg are shown in Table 1. The specimens were cut along 0° fiber direction with 250 mm in length, 25 mm in width and 1.48 mm in thickness as shown in Figure 1. The micro-scale structure of T800 prepreg is shown in Figure 2a. Each fiber-rich layer was spaced with a resin layer with a respective size of about 0.155 mm and 0.03 mm in the thickness direction as displayed in Figure 2b. The zone between each fiber was fully filled by the resin and the fibers were distributed randomly.

According to the ASTM D 3039-2008 standard, the quasi-static tensile tests were performed on the MTS-810 testing system (Mechanical Testing & Simulation, Eden Prairie, MN, USA) in Figure 1a. The averaged surface strain at the middle position of each specimen was recorded with a loading rate of 2 mm/min. Each damage morphology is shown in Figure 1b and its damage procedure can be illustrated obviously from the tensile stress–strain curve of each specimen in Figure 1c. In the loading process, the micro-scale cracks in the inter/intra-laminar area were firstly initiated at a strain of $\bar{\varepsilon }=0.19\%$. Then the matrix impregnated fibers become split, peel or pull out. The specimen finally fractured at $\bar{\varepsilon }=1.25\%$ with the failure modes of delamination being between layer-to-layer debonding and brittle fiber breakage. The initial averaged tensile modulus and ultimate strength are about $E_{exp}=65.1\;\mathrm{GPa}$ and $\sigma_{exp}=725\;\mathrm{MPa}$.

## 3. Numerical Analysis

### 3.1. Multi-Scale RVEs

For the interior structure in the experimental specimen as shown in Figure 2a,b, the Multi-scale RVEs were established, respectively, based on the principle of equivalent fiber volume and the intra/inter-phase fiber layer construction as displayed in Figure 2d–f. The interior fiber-packed patterns in each layer were distributed in Figure 2a and the corresponding interfacial phase was shown in Figure 2b. The paralleled fiber-rich layers were packed periodically in thickness with an inter-laminar zone filled with resin. Firstly, the multi-layer meso-RVE (in Figure 2c) was built according to the periodic array (eight layers) and real size (1.48 mm) of the angle-ply laminate as shown in Figure 2b. The thickness size of one layer in Figure 2e was divided by about 0.185 mm (relatively, 0.155 mm for fiber-rich layer and 0.03 mm for pure resin layer). The macroscale RVE of the angle-ply laminate was constructed with a stacking sequence of [+45/−45/0/90]_s_ of eight layers in Figure 2f. The sizes of macroscale RVE and mesoscale RVE in-plane were, respectively, adapted with 8 mm × 8 mm (x and y directions) × 1.48 mm (in the thickness direction) and 0.75 mm × 1.5 mm (x and y directions) × 0.185 mm (in the thickness direction). Secondly, the micro-RVE with a hexagonal fiber-packed pattern in the interior zone of each layer was obtained based on the concept of fiber volume between experimental and numerical models. The equivalent fiber volume fraction in the micro-RVE was calculated as 68.39% based on the ratio of total fiber volume in angle-ply laminate (57.3%) to the matrix impregnated fiber-rich zone volume fraction (83.78%) in the meso-RVE. Using a hexagonal fiber-packed distribution, the size of micro-RVE was determined with 17.971 mm (in length) × 12.71 mm (in width) × 10.0 mm (in fiber direction) assuming that the radius of fiber was 5 mm (*R* = 5 mm) as shown in Figure 2d.

**Figure 3:**
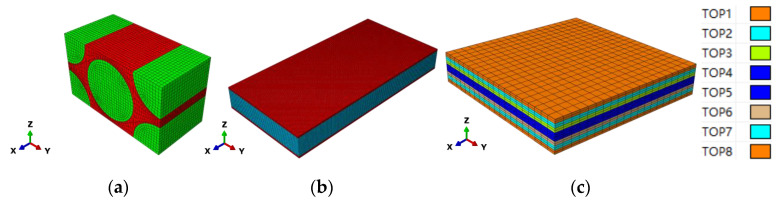
Multi-scale FE models of angle-ply laminate: (**a**) Micro-FE model; (**b**) Meso-FE model; (**c**) Macro-FE model.

The adapted FE (finite element) models are displayed in Figure 3. In the FE models, the x direction is the fiber direction in the micro/meso-FE models and the fiber/loading direction in the macro-FE model. The y direction is the in-plane transverse direction. The z direction is the thickness direction. The macro-FE model in Figure 3c was layered from Top 1 to Top 8 corresponding with the sequence of [+45/−45/0/90/90/0/−45/+45]. The C3D8R element was adopted in the micro-RVE with 25,040 elements, the meso-RVE with 213,750 elements, the macro-RVE with 6400 elements packed two elements in the thickness layer. In the multi-RVEs analysis, the periodic boundary conditions (PBCs) by master-slave node technology, the displacement loadings application and homogenized strategy from micro-FE model to meso-FE model, macro-FE model could be referenced to the detailed statements [29,38].

### 3.2. Multi-Scale Damage Models

The smear crack was proposed firstly in the application of the concrete fracture [39,40] and further extended in terms of composites analysis [41,42], The fully developed multi-scale damage models on the concept of smear cracks were provided based on the improvement of our previous research [27,28], such as pure shear and normal-shear coupled co-existing failure criteria. As shown in Figure 2(1)–(3), the micro-RVE was predicted with elastic constitutive relations from the primary mechanical constants as listed in Table 1, the maximum tensile, compressive principal stress and maximum shear stress failure criteria were applied as shown in Figure 2g. Once any failure criterion was satisfied in the material point of micro-RVE, the virtual smear crack would occur with corresponding stress components and the stiffness degrades quickly. In the meso-RVE analysis as displayed in Figure 2h, the fiber-rich zone was defined with the transversally isotropic model. In the fiber-rich zone, complex smear crack modes were incorporated independently with (1) single-smear crack initiated only by normal or shear loading failure, (2) double-smear cracks initiated by single-normal/shear combined loadings failures and (3) multi-smear cracks initiated by more single-normal/shear combined loadings failure. For each iteration in material point, the above-mentioned crack modes would occur when any longitudinal, transverse, or shear ultimate strength threshold was reached. Then the meso-RVE would lose the ability to undertake the corresponding loadings in particular directions, leading to different damage modes due to the different loading procedures. Similarly, an orthogonal elastic model with complex damage models could be applied in the analysis of macro-RVE as displayed in Figure 2i. The averaged properties with failure strengths of micro/meso/macro-RVEs could be homogenized by the application of periodic boundary conditions (PBC) as stated previously [28]. Moreover, the homogenized parameters were transferred with element properties from the current-scale model to the next one, such as micro-RVE, meso-RVE to macro-RVE.

A post-damage constitutive model to characterize the above-mentioned crack modes was established in the multi-scale damage analysis, to feature the cracks-initiated procedure upon progressive loading. In the loading process, the components in RVE undertake different elastic loads due to the structural distinction. Then a smear crack occurred once any one of the stress components in the material point of an element reached the failure criterion threshold. For example, a crack whose direction was perpendicular to the principal/normal directions in Figure 2g–i, these crack modes behave differently, dominated by the current stress states, such as pure tensile, compressive, or shear failure in the micro-RVE, the pure tensile, compressive, shear and their combinations failures in the meso/macro-RVEs. For the same material point in multi-RVEs, the existing and current initiated smear cracks were operated independently with the degradation of respective stress and stiffness components in further integration. For example, in Figure 2g, once a crack occurred in the plane perpendicular to the local principal direction 1, the element would lose the capacity of load transferred in the cracked plane, i.e., the stress components, $\sigma_{11},\tau_{12}$ and $\tau_{13}$ towards zeros, while the material point can still take other stress components ($\sigma_{22},\sigma_{33}$ and $\tau_{23}$) normally [27]. An isotropic post-damage model for fiber and resin in micro/meso-RVEs was presented in the local principal coordinate system with:

(1) $${\left\{\Delta \sigma \right\}}^{cr}=[D]{\left\{\Delta \varepsilon \right\}}^{cr}-\chi [B]{\left\{\sigma \right\}}^{cr}$$

Or in a full form as:

(2) $${\left\{\begin{array}{c} \Delta \sigma_{11}\\ \Delta \sigma_{22}\\ \Delta \sigma_{33}\\ \Delta \sigma_{12}\\ \Delta \sigma_{13}\\ \Delta \sigma_{23} \end{array}\right\}}^{cr}=\left[\begin{array}{cccccc} \beta Z_{1} & 0 & 0 & 0 & 0 & 0\\ 0 & Z_{1} & Z_{2} & 0 & 0 & 0\\ 0 & Z_{2} & Z_{1} & 0 & 0 & 0\\ 0 & 0 & 0 & \beta Z_{3} & 0 & 0\\ 0 & 0 & 0 & 0 & \beta Z_{3} & 0\\ 0 & 0 & 0 & 0 & 0 & Z_{3} \end{array}\right]{\left\{\begin{array}{c} \Delta \varepsilon_{11}\\ \Delta \varepsilon_{22}\\ \Delta \varepsilon_{33}\\ \Delta \varepsilon_{12}\\ \Delta \varepsilon_{13}\\ \Delta \varepsilon_{23} \end{array}\right\}}^{cr}-\chi \left[\begin{array}{cccccc} 1 & 0 & 0 & 0 & 0 & 0\\ 0 & 0 & 0 & 0 & 0 & 0\\ 0 & 0 & 0 & 0 & 0 & 0\\ 0 & 0 & 0 & 1 & 0 & 0\\ 0 & 0 & 0 & 0 & 1 & 0\\ 0 & 0 & 0 & 0 & 0 & 0 \end{array}\right]{\left\{\begin{array}{c} \sigma_{11}\\ \sigma_{22}\\ \sigma_{33}\\ \sigma_{12}\\ \sigma_{13}\\ \sigma_{23} \end{array}\right\}}^{cr}$$

where:

(3) $$Z_{1}=\frac{(1-\nu )E}{\left(1+\nu )(1-2\nu \right)},Z_{2}=\frac{\nu E}{\left(1+\nu )(1-2\nu \right)},Z_{3}=\frac{E}{2(1+\nu )}$$

In the local coordinate system, β (e.g., $\beta =10^{-3}\times \Delta t$) and χ (e.g., χ=0.4) indicates the stiffness and stress components degradation factors in these three directions. Once a crack occurred, the parameters of $\beta Z_{1}$ and $\beta Z_{2}$ would be degraded quickly in the following iterations, meaning the stress increments, $\beta Z_{1}\Delta \varepsilon_{11}$,$\;\beta Z_{3}\Delta \varepsilon_{12}$ and $\beta Z_{3}\Delta \varepsilon_{13}$ were suppressed by a small value. For the stress components, $\sigma_{11},\sigma_{12}\;\mathrm{and}\;\sigma_{13}$, they degrade by the ratio of 0.4. Upon further loading, another smear crack perpendicular to 2 direction in local system was created in the above-mentioned same material point as stated in Equation (2). The progressive post-damage model could be expressed in Equation (4), which means only the stress (3) undertakes loadings normally. This material point loses the ability to undertake loadings fully once the smear crack is initiated with its surface perpendicular to normal direction 3 as shown in Equation (5). Finally, the material point failed absolutely with multiple cracks co-occurring as displayed in Equations (1)–(4) in the following FE iterations.

(4) $${\left\{\begin{array}{c} \Delta \sigma_{11}\\ \Delta \sigma_{22}\\ \Delta \sigma_{33}\\ \Delta \sigma_{12}\\ \Delta \sigma_{13}\\ \Delta \sigma_{23} \end{array}\right\}}^{cr}=\left[\begin{array}{cccccc} \beta Z_{1} & 0 & 0 & 0 & 0 & 0\\ 0 & \beta Z_{1} & 0 & 0 & 0 & 0\\ 0 & 0 & Z_{1} & 0 & 0 & 0\\ 0 & 0 & 0 & \beta Z_{3} & 0 & 0\\ 0 & 0 & 0 & 0 & \beta Z_{3} & 0\\ 0 & 0 & 0 & 0 & 0 & \beta Z_{3} \end{array}\right]{\left\{\begin{array}{c} \Delta \varepsilon_{11}\\ \Delta \varepsilon_{22}\\ \Delta \varepsilon_{33}\\ \Delta \varepsilon_{12}\\ \Delta \varepsilon_{13}\\ \Delta \varepsilon_{23} \end{array}\right\}}^{cr}-\chi \left[\begin{array}{cccccc} 1 & 0 & 0 & 0 & 0 & 0\\ 0 & 1 & 0 & 0 & 0 & 0\\ 0 & 0 & 0 & 0 & 0 & 0\\ 0 & 0 & 0 & 1 & 0 & 0\\ 0 & 0 & 0 & 0 & 1 & 0\\ 0 & 0 & 0 & 0 & 0 & 1 \end{array}\right]{\left\{\begin{array}{c} \sigma_{11}\\ \sigma_{22}\\ \sigma_{33}\\ \sigma_{12}\\ \sigma_{13}\\ \sigma_{23} \end{array}\right\}}^{cr}$$

(5) $${\left\{\begin{array}{c} \Delta \sigma_{11}\\ \Delta \sigma_{22}\\ \Delta \sigma_{33}\\ \Delta \sigma_{12}\\ \Delta \sigma_{13}\\ \Delta \sigma_{23} \end{array}\right\}}^{cr}=\left[\begin{array}{cccccc} \beta Z_{1} & 0 & 0 & 0 & 0 & 0\\ 0 & \beta Z_{1} & 0 & 0 & 0 & 0\\ 0 & 0 & \beta Z_{1} & 0 & 0 & 0\\ 0 & 0 & 0 & \beta Z_{3} & 0 & 0\\ 0 & 0 & 0 & 0 & \beta Z_{3} & 0\\ 0 & 0 & 0 & 0 & 0 & \beta Z_{3} \end{array}\right]{\left\{\begin{array}{c} \Delta \varepsilon_{11}\\ \Delta \varepsilon_{22}\\ \Delta \varepsilon_{33}\\ \Delta \varepsilon_{12}\\ \Delta \varepsilon_{13}\\ \Delta \varepsilon_{23} \end{array}\right\}}^{cr}-\chi \left[\begin{array}{cccccc} 1 & 0 & 0 & 0 & 0 & 0\\ 0 & 1 & 0 & 0 & 0 & 0\\ 0 & 0 & 1 & 0 & 0 & 0\\ 0 & 0 & 0 & 1 & 0 & 0\\ 0 & 0 & 0 & 0 & 1 & 0\\ 0 & 0 & 0 & 0 & 0 & 1 \end{array}\right]{\left\{\begin{array}{c} \sigma_{11}\\ \sigma_{22}\\ \sigma_{33}\\ \sigma_{12}\\ \sigma_{13}\\ \sigma_{23} \end{array}\right\}}^{cr}$$

For the fiber-rich zone in the middle layer of the meso-RVE as shown in Figure 2e,h, the transversely isotropic post-damage model has been compiled in the UMAT subroutine with single, double and complex smear crack modes. For a single smear crack in the fiber-reinforced zone, such as the fiber fracture and matrix crack. For example, when the fiber fractured in the fiber direction with crack 1 in Figure 2h, its transversely isotropic post-damage constitutive model for the fiber-rich zone (in Figure 2e) can be given in Equation (6) [27].

(6) $${\left\{\begin{array}{c} \Delta \sigma_{11}\\ \Delta \sigma_{22}\\ \Delta \sigma_{33}\\ \Delta \sigma_{12}\\ \Delta \sigma_{13}\\ \Delta \sigma_{23} \end{array}\right\}}^{cr}=\left[\begin{array}{cccccc} \beta C_{1} & 0 & 0 & 0 & 0 & 0\\ 0 & C_{2} & C_{3} & 0 & 0 & 0\\ 0 & C_{3} & C_{2} & 0 & 0 & 0\\ 0 & 0 & 0 & \beta C_{4} & 0 & 0\\ 0 & 0 & 0 & 0 & \beta C_{4} & 0\\ 0 & 0 & 0 & 0 & 0 & C_{6} \end{array}\right]{\left\{\begin{array}{c} \Delta \varepsilon_{11}\\ \Delta \varepsilon_{22}\\ \Delta \varepsilon_{33}\\ \Delta \varepsilon_{12}\\ \Delta \varepsilon_{13}\\ \Delta \varepsilon_{23} \end{array}\right\}}^{cr}-\chi \left[\begin{array}{cccccc} 1 & 0 & 0 & 0 & 0 & 0\\ 0 & 0 & 0 & 0 & 0 & 0\\ 0 & 0 & 0 & 0 & 0 & 0\\ 0 & 0 & 0 & 1 & 0 & 0\\ 0 & 0 & 0 & 0 & 1 & 0\\ 0 & 0 & 0 & 0 & 0 & 0 \end{array}\right]{\left\{\begin{array}{c} \sigma_{11}\\ \sigma_{22}\\ \sigma_{33}\\ \sigma_{12}\\ \sigma_{13}\\ \sigma_{23} \end{array}\right\}}^{cr}$$

where:

(7) $$\begin{array}{l} C_{1}=\frac{E_{11}(1-\nu_{23}^{2})}{1-\nu_{23}^{2}-2(1+\nu_{23})\frac{E_{22}}{E_{11}}\nu_{12}^{2}},C_{2}=\frac{E_{22}(1-\frac{E_{22}}{E_{11}}\nu_{12}^{2})}{1-\nu_{23}^{2}-2(1+\nu_{23})\frac{E_{22}}{E_{11}}\nu_{12}^{2}}\\ C_{3}=\frac{E_{22}(\nu_{23}+\frac{E_{22}}{E_{12}}\nu_{12}^{2})}{1-\nu_{23}^{2}-2(1+\nu_{23})\frac{E_{22}}{E_{11}}\nu_{12}^{2}},C_{4}=G_{12},C_{6}=\frac{E_{22}}{2(1+\nu_{23})} \end{array}$$

*E*_11_ and *E*_22_ are the modulus in the fiber direction and transverse direction, respectively.

In the continuous loading in the meso-RVE analysis, the double smear crack modes with new normal-loading induced cracks or new shear-loading induced cracks. Assuming crack 2 in Figure 2h occurred in the coming iteration, the progressive damage could be described in Equation (8). The fully damaged failures were realized in Equation (9), such as the 3-directional crack occurring. Up to the current iteration in the meso-RVE analysis, the material point would never undertake any loadings with the stress components and stiffness being degraded to a small value, leaving the damaged modes with fiber fractures and matrix cracks.

(8) $${\left\{\begin{array}{c} \Delta \sigma_{11}\\ \Delta \sigma_{22}\\ \Delta \sigma_{33}\\ \Delta \sigma_{12}\\ \Delta \sigma_{13}\\ \Delta \sigma_{23} \end{array}\right\}}^{cr}=\left[\begin{array}{cccccc} \beta C_{1} & 0 & 0 & 0 & 0 & 0\\ 0 & \beta C_{2} & 0 & 0 & 0 & 0\\ 0 & 0 & C_{2} & 0 & 0 & 0\\ 0 & 0 & 0 & \beta C_{4} & 0 & 0\\ 0 & 0 & 0 & 0 & \beta C_{4} & 0\\ 0 & 0 & 0 & 0 & 0 & \beta C_{6} \end{array}\right]{\left\{\begin{array}{c} \Delta \varepsilon_{11}\\ \Delta \varepsilon_{22}\\ \Delta \varepsilon_{33}\\ \Delta \varepsilon_{12}\\ \Delta \varepsilon_{13}\\ \Delta \varepsilon_{23} \end{array}\right\}}^{cr}-\chi \left[\begin{array}{cccccc} 1 & 0 & 0 & 0 & 0 & 0\\ 0 & 1 & 0 & 0 & 0 & 0\\ 0 & 0 & 0 & 0 & 0 & 0\\ 0 & 0 & 0 & 1 & 0 & 0\\ 0 & 0 & 0 & 0 & 1 & 0\\ 0 & 0 & 0 & 0 & 0 & 1 \end{array}\right]{\left\{\begin{array}{c} \sigma_{11}\\ \sigma_{22}\\ \sigma_{33}\\ \sigma_{12}\\ \sigma_{13}\\ \sigma_{23} \end{array}\right\}}^{cr}$$

(9) $${\left\{\begin{array}{c} \Delta \sigma_{11}\\ \Delta \sigma_{22}\\ \Delta \sigma_{33}\\ \Delta \sigma_{12}\\ \Delta \sigma_{13}\\ \Delta \sigma_{23} \end{array}\right\}}^{cr}=\left[\begin{array}{cccccc} \beta C_{1} & 0 & 0 & 0 & 0 & 0\\ 0 & \beta C_{2} & 0 & 0 & 0 & 0\\ 0 & 0 & \beta C_{2} & 0 & 0 & 0\\ 0 & 0 & 0 & \beta C_{4} & 0 & 0\\ 0 & 0 & 0 & 0 & \beta C_{4} & 0\\ 0 & 0 & 0 & 0 & 0 & \beta C_{6} \end{array}\right]{\left\{\begin{array}{c} \Delta \varepsilon_{11}\\ \Delta \varepsilon_{22}\\ \Delta \varepsilon_{33}\\ \Delta \varepsilon_{12}\\ \Delta \varepsilon_{13}\\ \Delta \varepsilon_{23} \end{array}\right\}}^{cr}-\chi \left[\begin{array}{cccccc} 1 & 0 & 0 & 0 & 0 & 0\\ 0 & 1 & 0 & 0 & 0 & 0\\ 0 & 0 & 1 & 0 & 0 & 0\\ 0 & 0 & 0 & 1 & 0 & 0\\ 0 & 0 & 0 & 0 & 1 & 0\\ 0 & 0 & 0 & 0 & 0 & 1 \end{array}\right]{\left\{\begin{array}{c} \sigma_{11}\\ \sigma_{22}\\ \sigma_{33}\\ \sigma_{12}\\ \sigma_{13}\\ \sigma_{23} \end{array}\right\}}^{cr}$$

Regarding the macro-RVE as shown in Figure 2f, an orthogonal anisotropic post-damage model was incorporated, such as the delamination, fiber fracture and matrix crack, etc. For example, crack 1 was initiated with its surface perpendicular to the x direction as shown in Figure 2i, its post-damage constitutive relation can be defined in Equation (10) [27].

(10) $${\left\{\begin{array}{c} \Delta \sigma_{11}\\ \Delta \sigma_{22}\\ \Delta \sigma_{33}\\ \Delta \sigma_{12}\\ \Delta \sigma_{13}\\ \Delta \sigma_{23} \end{array}\right\}}^{cr}=\left[\begin{array}{cc} \left[L\right] & \begin{array}{ccc} 0 & 0 & 0\\ 0 & 0 & 0\\ 0 & 0 & 0 \end{array}\\ \begin{array}{ccc} 0 & 0 & 0\\ 0 & 0 & 0\\ 0 & 0 & 0 \end{array} & \left[M\right] \end{array}\right]{\left\{\begin{array}{c} \Delta \varepsilon_{11}\\ \Delta \varepsilon_{22}\\ \Delta \varepsilon_{33}\\ \Delta \varepsilon_{12}\\ \Delta \varepsilon_{13}\\ \Delta \varepsilon_{23} \end{array}\right\}}^{cr}-\chi \left[\begin{array}{cccccc} 1 & 0 & 0 & 0 & 0 & 0\\ 0 & 0 & 0 & 0 & 0 & 0\\ 0 & 0 & 0 & 0 & 0 & 0\\ 0 & 0 & 0 & 1 & 0 & 0\\ 0 & 0 & 0 & 0 & 1 & 0\\ 0 & 0 & 0 & 0 & 0 & 0 \end{array}\right]{\left\{\begin{array}{c} \sigma_{11}\\ \sigma_{22}\\ \sigma_{33}\\ \sigma_{12}\\ \sigma_{13}\\ \sigma_{23} \end{array}\right\}}^{cr}$$

where:

(11) $$\begin{array}{l} \left[L\right]=\frac{1}{H}\left[\begin{array}{ccc} \beta E_{11}(1-E_{33}/E_{22}\nu_{23}^{2}) & 0 & 0\\ 0 & E_{22}(1-E_{33}/E_{11}\nu_{13}^{2}) & E_{33}(\nu_{23}+E_{22}/E_{11}\nu_{12}\nu_{13})\\ 0 & E_{33}(\nu_{23}+E_{22}/E_{11}\nu_{12}\nu_{13}) & E_{33}(1-E_{22}/E_{11}\nu_{12}^{2}) \end{array}\right]\\ \left[M\right]=\left[\begin{array}{ccc} \beta G_{12} & 0 & 0\\ 0 & \beta G_{13} & 0\\ 0 & 0 & G_{23} \end{array}\right]\\ H=\frac{E_{11}E_{22}E_{33}-\nu_{23}^{2}E_{11}E_{33}^{2}-\nu_{12}^{2}E_{22}^{2}E_{33}-2\nu_{12}\nu_{13}\nu_{23}E_{22}E_{33}^{2}-\nu_{13}^{2}E_{22}E_{33}^{2}}{E_{11}E_{22}E_{33}} \end{array}$$

Once crack 1 was initiated as listed in Equation (10), the continuous damage still can occur with crack 2 and 3 with further loadings by expression Equations (12) and (13).

(12) $$\begin{array}{l} \left[L\right]=\frac{1}{H}\left[\begin{array}{ccc} \beta E_{11}(1-E_{33}/E_{22}\nu_{23}^{2}) & 0 & 0\\ 0 & \beta E_{22}(1-E_{33}/E_{11}\nu_{13}^{2}) & 0\\ 0 & 0 & E_{33}(1-E_{22}/E_{11}\nu_{12}^{2}) \end{array}\right]\\ \left[M\right]=\left[\begin{array}{ccc} \beta G_{12} & 0 & 0\\ 0 & \beta G_{13} & 0\\ 0 & 0 & \beta G_{23} \end{array}\right] \end{array}$$

(13) $$\begin{array}{l} \left[L\right]=\frac{1}{H}\left[\begin{array}{ccc} \beta E_{11}(1-E_{33}/E_{22}\nu_{23}^{2}) & 0 & 0\\ 0 & \beta E_{22}(1-E_{33}/E_{11}\nu_{13}^{2}) & 0\\ 0 & 0 & \beta E_{33}(1-E_{22}/E_{11}\nu_{12}^{2}) \end{array}\right]\\ \left[M\right]=\left[\begin{array}{ccc} \beta G_{12} & 0 & 0\\ 0 & \beta G_{13} & 0\\ 0 & 0 & \beta G_{23} \end{array}\right] \end{array}$$

The above-mentioned damage and post-damage responses analysis of multi-RVE were defined in the local coordinate system. Equation (1) would be transformed to the global coordinate system while the local coordinate system was different from the global coordinate system in FE analyses:

(14) $${\left\{\Delta \sigma \right\}}^{gl}=[D^{\prime }]{\left\{\Delta \varepsilon \right\}}^{gl}-\chi [B^{\prime }]{\left\{\sigma \right\}}^{gl}$$

where:

(15) $$\begin{array}{l} \left[D^{\prime }]={\left[T\right]}^{T}[D][T\right]\\ \left[B^{\prime }]={\left[T\right]}^{T}[B][T\right]\\ {}[T]=\left[\begin{array}{cccccc} l_{1}^{2} & m_{1}^{2} & n_{1}^{2} & l_{1}m_{1} & m_{1}n_{1} & n_{1}l_{1}\\ l_{2}^{2} & m_{2}^{2} & n_{2}^{2} & l_{2}m_{2} & m_{2}n_{2} & n_{2}l_{2}\\ l_{3}^{2} & m_{3}^{2} & n_{3}^{2} & l_{3}m_{3} & m_{3}n_{3} & n_{3}l_{3}\\ 2l_{1}l_{2} & 2m_{1}m_{2} & 2n_{1}n_{2} & l_{1}m_{2}+l_{2}m_{1} & m_{1}n_{2}+m_{2}n_{1} & n_{1}l_{2}+n_{2}l_{1}\\ 2l_{2}l_{3} & 2m_{2}m_{3} & 2n_{2}n_{3} & l_{2}m_{3}+l_{3}m_{2} & m_{2}n_{3}+m_{3}n_{2} & n_{2}l_{3}+n_{3}l_{2}\\ 2l_{3}l_{1} & 2m_{3}m_{1} & 2n_{3}n_{1} & l_{3}m_{1}+l_{1}m_{3} & m_{3}n_{1}+m_{1}n_{3} & n_{3}l_{1}+n_{1}l_{3} \end{array}\right] \end{array}$$

$l_{i},m_{i}\;\mathrm{and}\;n_{i}$ are directional cosines of the local coordinate axes in global coordinate system.

Simultaneously, the shear-dominated smear cracks were also incorporated in the multi-RVEs analysis. The *S*_12_-dominated crack led to the quick degradation of $\sigma_{11}$ $\sigma_{12}$ and $\sigma_{13}$, $E_{11},G_{12}$ and $G_{13}$ after a few iterations. For a progressive displacement increment loading iteration in multi-scale RVEs analysis, there would be multiple smear crack modes occurring in the same material point due to different loading procedures and levels, up to its full failure.

**Figure 4:**
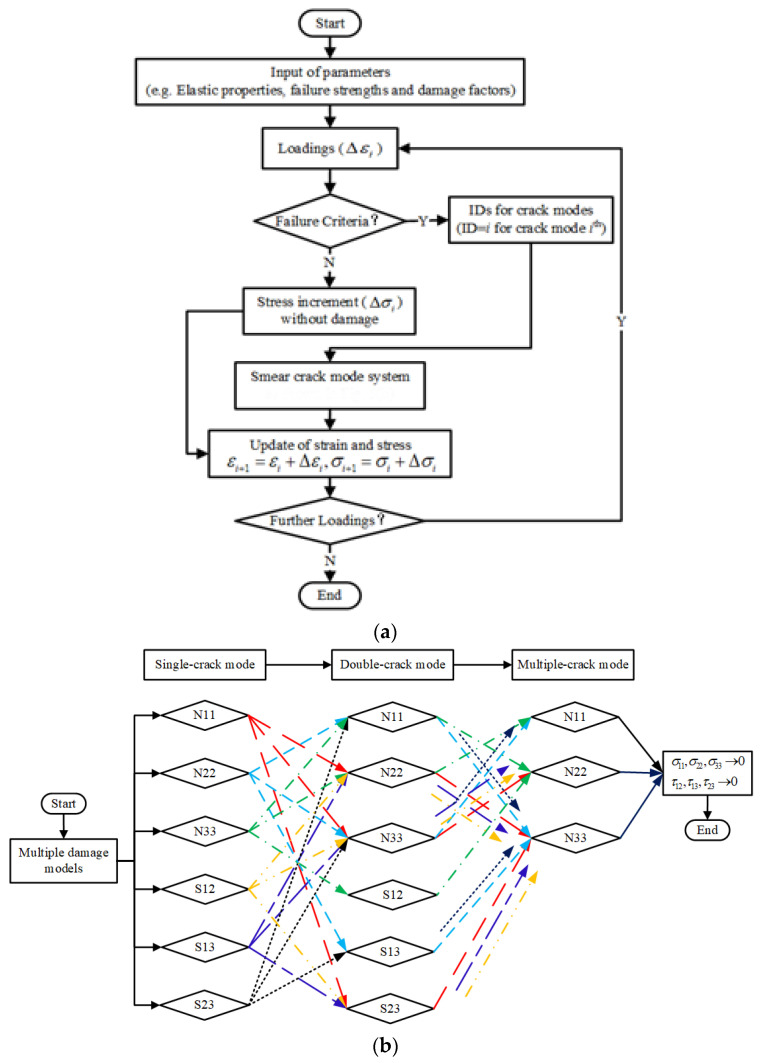
Damage and post-damage flowchart of user-defined material subroutines (UMATs): (**a**) UMAT flow chart; (**b**) Numerical damage path for different smear cracks.

In the multi-RVEs analysis, the above-mentioned damage and post-damage constitutive models were compiled into the user-defined material subroutines (UMATs) as shown in Figure 4a, including all potential kinds of damage modes in the full format as displayed in Figure 4b (*N_ii_* indicates smear crack surface to be paralleled with the *i*-directional normal loading damage. *S_ij_* indicates the smear crack damaged in *ij*-directional shear loading). For initial loadings, the multi-RVEs behave elastically and then were damaged progressively, such as from the single-crack mode, double-crack mode to multiple-crack mode, or partially. Once a material point in multi-RVEs was cracked on the principle of the maximum principle/tensile, minimum principle/compressive strengths and maximum shear stress failure criteria as shown in Figure 2g–i. The above-mentioned post-damage mechanisms, such as single-crack, double-crack and multi-crack modes were performed independently upon further applied loadings and this made the multi-RVEs lose the ability to undertake loadings both in the particular directions at the single-crack or double-crack stage and in all directions at the multiple-crack stage. Finally, multi-RVEs UMATs were combined with ABAQUS/Standard, to obtain global and local responses with their damage mechanism of multi-scale RVEs, respectively.

## 4. Results and Discussions

### 4.1. Global/Local Responses of Micro-RVE

**Figure 5:**
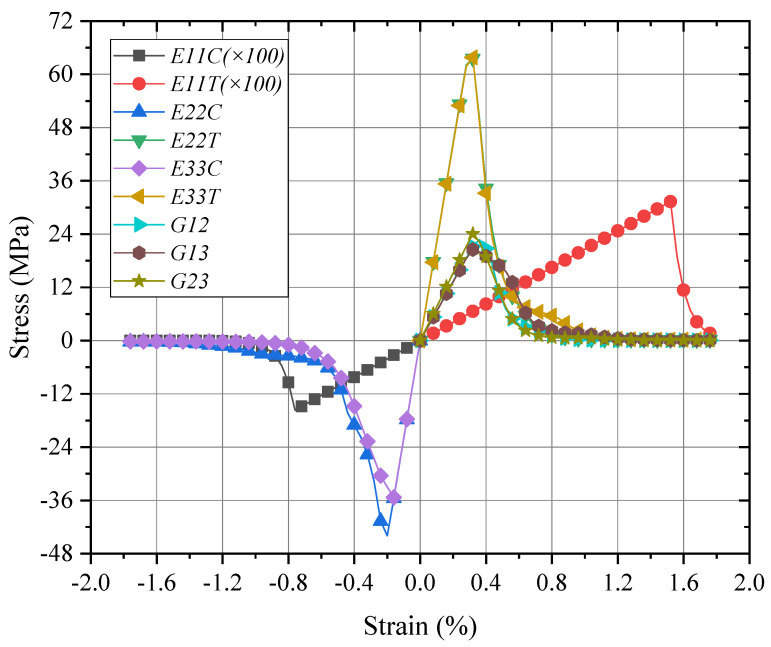
Global responses of micro-RVE under pure normal/shear loadings.

Based on the properties of matrix and fiber in Table 1, the mechanical responses with failure strengths of micro-RVE in Figure 3a are predicted under six independent pure normal/shear loadings as shown in Figure 5 (Note: 1-fiber direction and 2, 3-transverse directions. *ET, EC* and *G* identify tensile, compressive elongation and shear loading cases, respectively. *E11T* × 100 means its value magnified by 100 times). The micro-RVE behaves transversely isotropic with a much higher tensile modulus along the fiber direction (*E*_11_ = 206.0 GPa) than transversely (*E*_22_ = 22.2 GPa, *E*_33_ = 22.1 GPa). The micro-RVE has the higher ultimate strength in the fiber direction ($\sigma_{1t}=3131.5\;\mathrm{MPa}\;\mathrm{and}\;\sigma_{1c}=-1568.8\;\mathrm{MPa}$) than transversely $\left(\sigma_{2t}=63.6\;\mathrm{MPa}\;\mathrm{and}\;\sigma_{2c}=-40.7\;\mathrm{MPa}\right)$ and $(\sigma_{3t}=63.8\;\mathrm{MPa}\;\mathrm{and}\;\sigma_{3c}=-30.3\;\mathrm{MPa}$). For each loading case, such as x-directional tensile loading (*E11T*), once the fiber fractured damage initiates at about $\varepsilon_{11}=1.5\%$, the micro-RVE behaves with a peak value and then degrades gradually quickly and loses its ability to endure further loadings in integrity. The mechanical homogenized parameters of micro-RVE are calculated and listed in Table 2.

**Figure 6:**
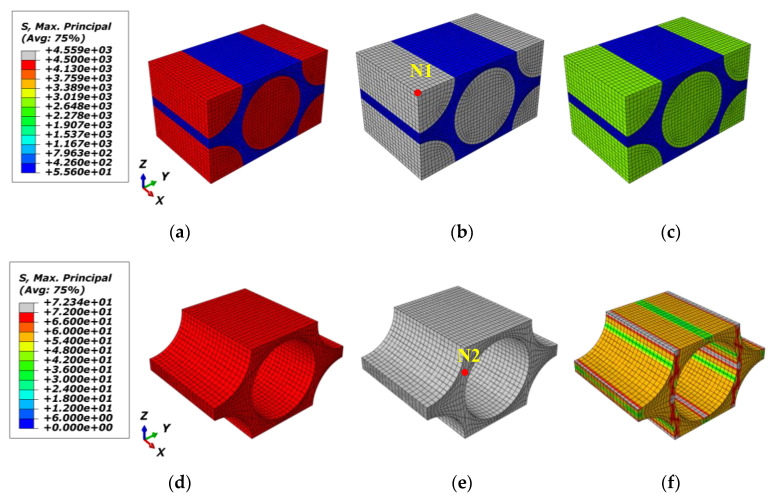
Damage initiation and evolution of micro-RVE under x direction tensile loading: (**a**) ${\bar{\varepsilon }}_{11}=1.48\%$; (**b**) ${\bar{\varepsilon }}_{11}=1.52\%$; (**c**) ${\bar{\varepsilon }}_{11}=1.56\%$; (**d**) ${\bar{\varepsilon }}_{11}=1.96\%$; (**e**) ${\bar{\varepsilon }}_{11}=2.00\%$; (**f**) ${\bar{\varepsilon }}_{11}=2.04\%$.

At the initial loading stage, the micro-RVE features the same response for tensile and compressive loadings in the same loading direction. Upon further loading, the cracks in fiber/resin are initiated once any of the predefined criteria in UMATs are reached under tensile, compressive or shear displacement loading. For the fiber-directional tensile loading case (*E11T*), regarding a fiber failure strength of $\sigma_{ft}=4.5\;\mathrm{GPa}$ and matrix failure strength of $\sigma_{mt}=72\;\mathrm{MPa}$ (in Table 1), the fiber area where the maximum principal stress exceeded the above failure threshold is changed into the grey zone as shown in Figure 6b,e. The further details are revealed with two local nodes selected as *N1* node in Figure 5 and *N2* node in Figure 6e.

**Figure 7:**
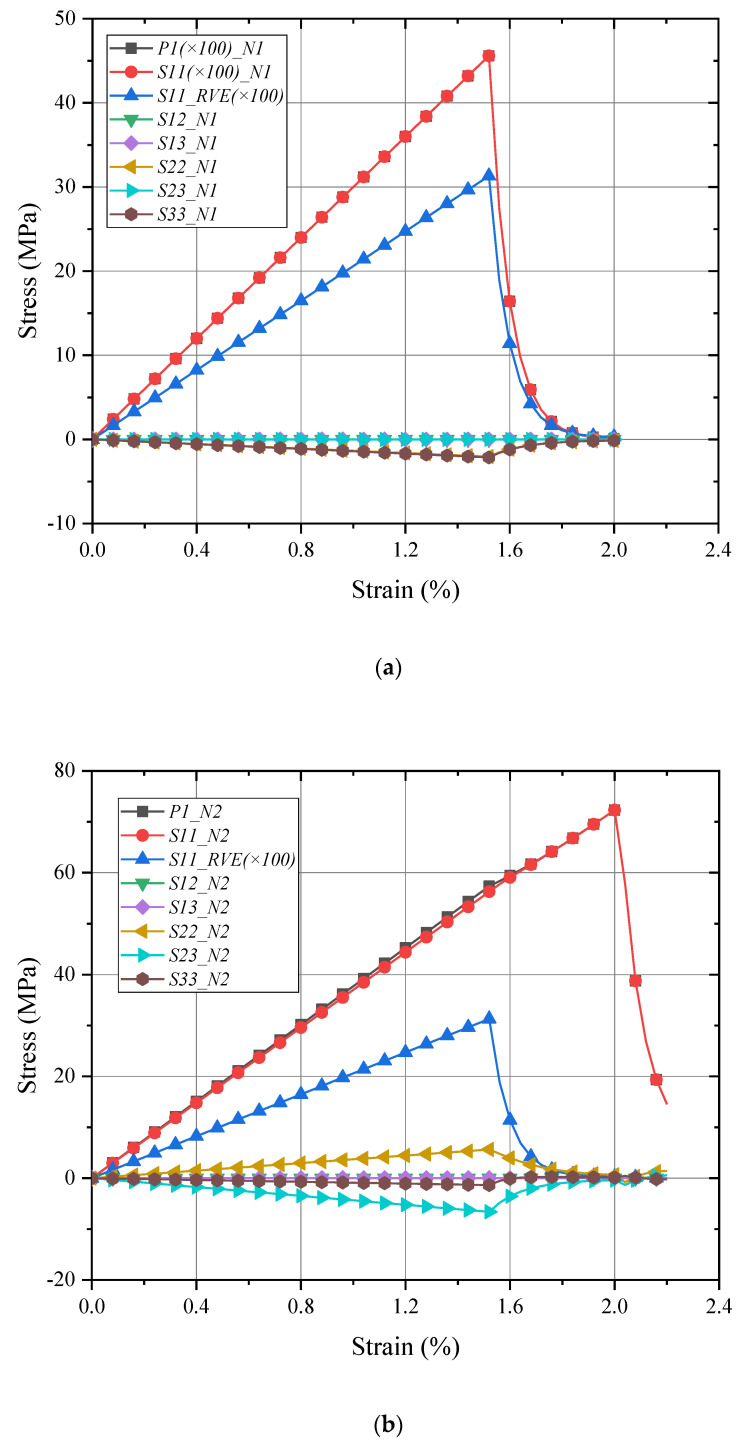
Local responses of micro-RVE under x direction tensile loading: (**a**) N1 in fiber; (**b**) N2 in matrix.

The local responses of fiber/matrix (*N1*/*N2*) in micro-RVE under fiber longitudinal loading are given in Figure 7. The maximum stresses in fiber and matrix are $S_{11\_f}=4.56\;\mathrm{GPa}$ (in Figure 6a) and $S_{11\_m}=72.3\;\mathrm{MPa}$ (in Figure 7b), respectively. They both keep enough accuracy for the predefined failure strength as listed in Table 1. The damaged stress components are degraded with a big decline in a few iterations once a crack has occurred, while other stress components are still set to be carried out normally without this effect.

### 4.2. Global/Local Responses of Meso-RVE

In the meso-RVE FE analysis, the middle layer of the fiber-rich zone in the meso-RVE (in Figure 3b) is idealized as the transversely isotropic properties in Table 2 and its surface layer has the isotropic property of resin in Table 1. Due to the interior element’s distinction in micro-RVE, the transverse properties of the fiber-rich layer are defined as a revision with $(E_{22}=E_{33}=21.1\;\mathrm{GPa},\;\sigma_{2t}=\sigma_{3t}=63.6\;\mathrm{Mpa},\;\sigma_{2c}=\sigma_{3c}=-33.3\;\mathrm{Mpa}$ and $\tau_{12}=\tau_{13}=20.5\;\mathrm{Mpa}$).

**Figure 8:**
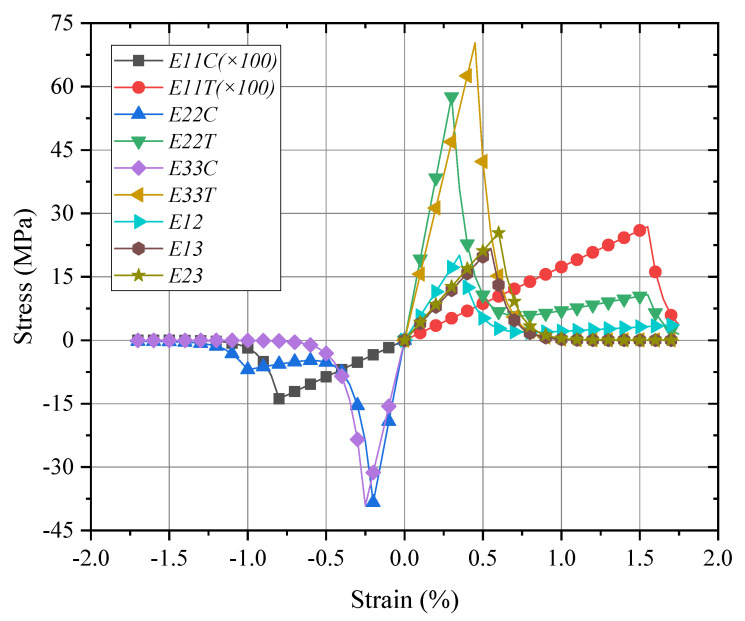
Global responses of meso-RVE under pure normal/shear loadings.

Due to the essential distinction in the structure of the real fiber-packed layer in the laminate, as shown in Figure 3b, a resin layer between each fiber layer makes the meso-RVE with different behavior characteristics in the transverse sections as shown in Figure 8 (e.g.,$E_{22}=19.2\;\mathrm{GPa},\sigma_{2t}=57.6\;\mathrm{MPa},\;\sigma_{2c}=-38.4\;\mathrm{MPa}$ and $E_{33}=15.6\;\mathrm{GPa},\;\sigma_{3t}=70.4\;\mathrm{MPa},\;\sigma_{3c}=-39.1\;\mathrm{MPa}$). The meso-RVE slightly features the excellent behavior in the thickness direction than in the other transverse directions. Comparatively, the meso-RVE in fiber direction has the higher modulus and failure strengths, such as $E_{11}=173.2\;\mathrm{GPa},\sigma_{1t}=2684.4\;\mathrm{MPa},\;\sigma_{1c}=-1385.5\;\mathrm{MPa}.$ The further numerical results are referenced in Table 3.

**Figure 9:**
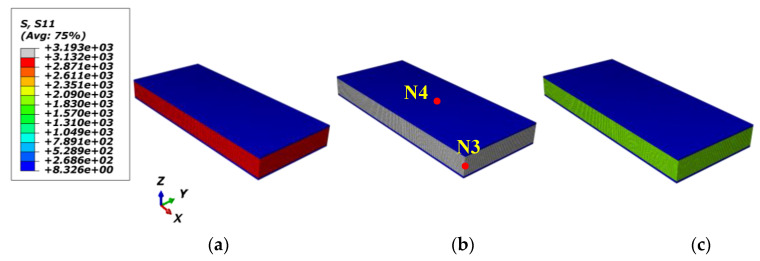
Damage initiation and evolution of meso-RVE under *x* direction tensile loading: (**a**) ${\bar{\varepsilon }}_{11}=1.50\%$; (**b**) ${\bar{\varepsilon }}_{11}=1.55\%$; (**c**) ${\bar{\varepsilon }}_{11}=$1.60%.

**Figure 10:**
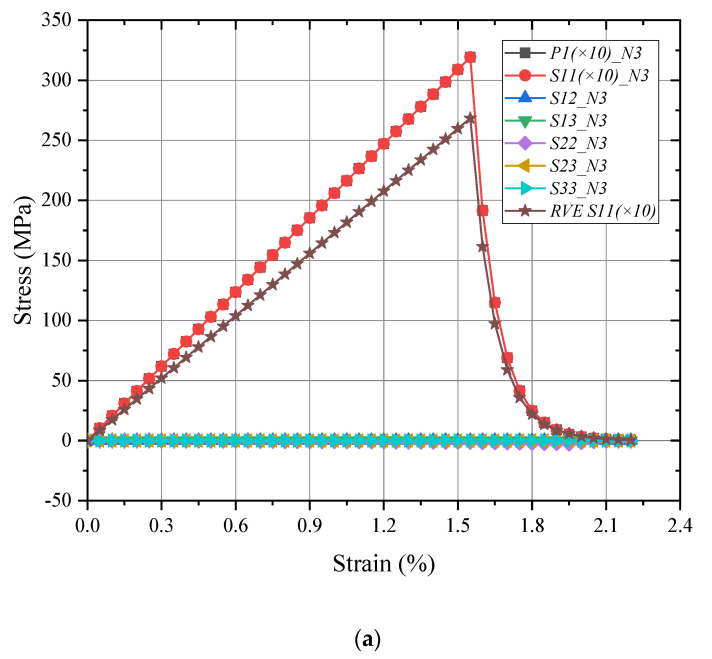

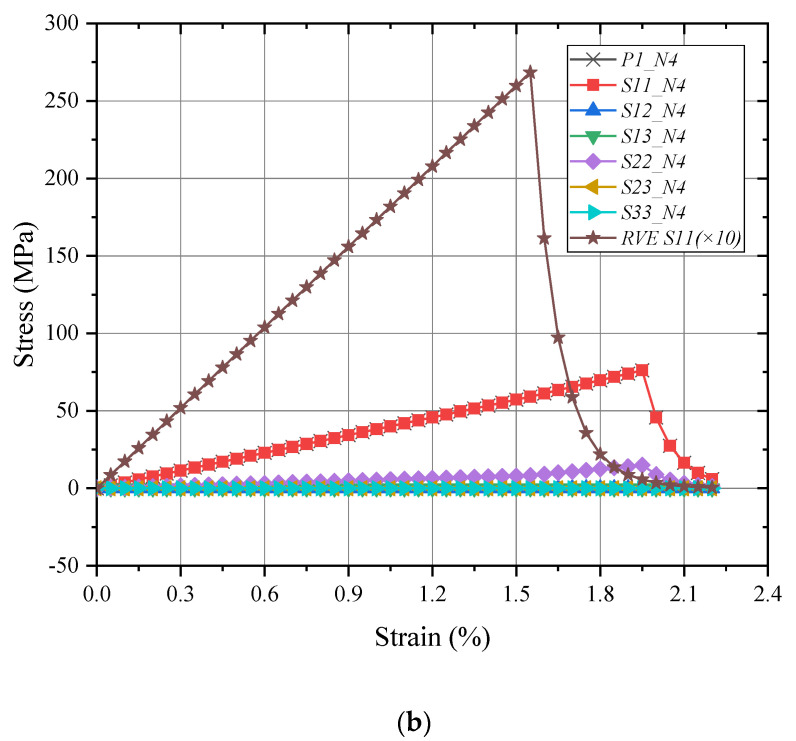
Local responses of meso-RVE under x direction tensile loading: (**a**) N3 in fiber-rich layer; (**b**) N4 in matrix layer.

For a detailed analysis in the meso-RVE, its local responses (N3 in fiber-rich zone and N4 in resin layer) are selected in Figure 9 and Figure 10. In the longitudinal loading process, local damage is initiated when the x direction stress component ($S_{11}=3193\;\mathrm{MPa}>\sigma_{1t}=3131.5\;\mathrm{MPa}$) reached the ranges of failure strengths listed in Table 2. Then the meso-RVE loses the ability to undertake continuous loading in integrity due to the fiber breakage (nearby $\varepsilon_{f}=1.5\%$) of fiber-rich layer, as shown in Figure 7a, and matrix cracking (nearby $\varepsilon_{m}=2.0\%$), as shown in Figure 6b.

### 4.3. Global/Local Responses of Macro-RVE

From the engineering parameters of the meso-RVE as listed in Table 3, the mechanical responses of macro-RVE (in Figure 3c) are analyzed and its mechanical parameters are listed in Table 4.

**Figure 11:**
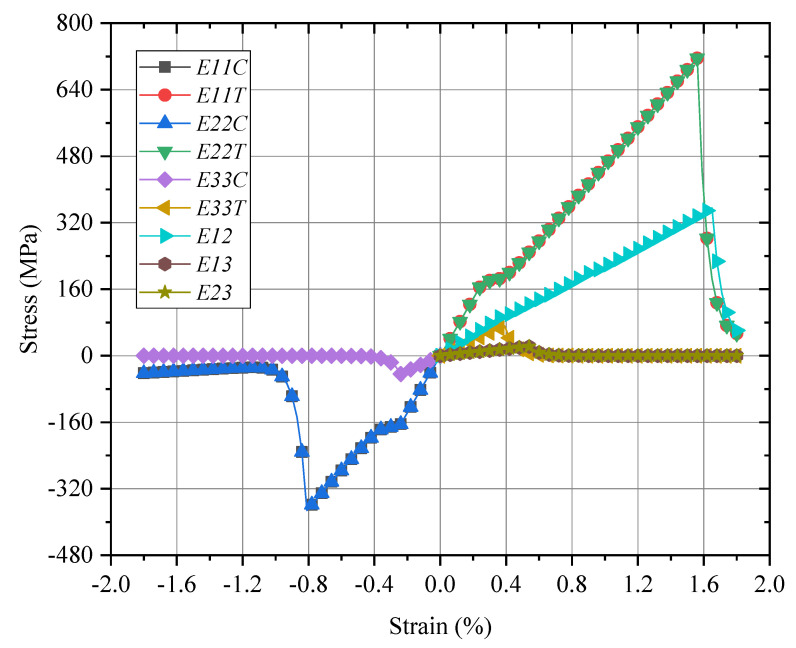
Global responses of macro-RVE under pure normal/shear loadings.

**Figure 12:**
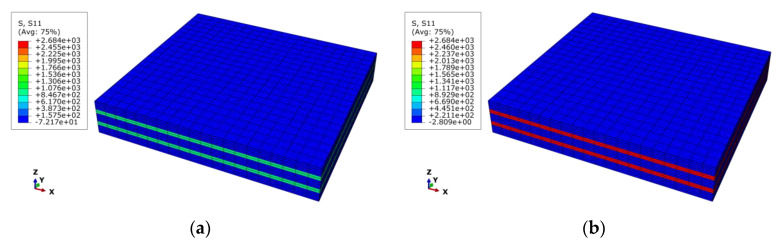

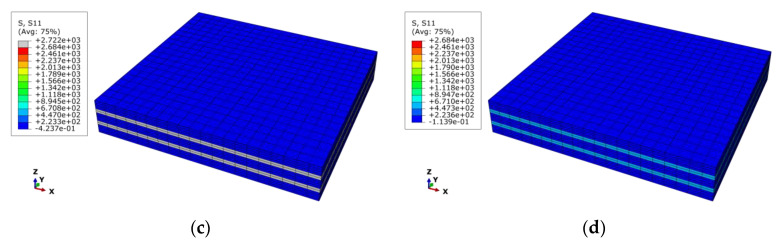
Fiber fracture evolution in macro-RVE under x direction tensile loading (*S*11): (**a**) ${\bar{\varepsilon }}_{11}=0.54\%$; (**b**) ${\bar{\varepsilon }}_{11}=$1.44%; (**c**) ${\bar{\varepsilon }}_{11}=$1.54%; (**d**) ${\bar{\varepsilon }}_{11}=1.68\%$.

The mechanical responses of the macro-RVE in normal, shear directions, respectively, are provided in Figure 11. In macro-RVE’s analysis, the laminar layer failure criteria, such as the tensile, compressive and shear failure strengths of meso-RVE, as shown in Table 3, are incorporated into the macro-RVE model independently. For example, under x direction tensile loading (*E*_11*T*_) with its damage evolution as shown in Figure 12, the tensile behaviors show linear responses at the initial stage ($\varepsilon_{macro}<0.3\%$) with a tensile modulus of $E_{macro}=68.6\;\mathrm{GPa}$. Nearby, the 0.3% stage of strain, the damaged zone is initiated possibly as delamination or matrix crack, the tensile modulus slightly reduces to $E_{macro}=45.6\;\mathrm{GPa}$. With further loadings, the macro-RVE continues to undertake the higher load in integrity and fracture due to fiber split and breaks at about $\varepsilon_{macro}=1.55\%$. Compared to the tested results of $E_{exp}=65.1\;\mathrm{GPa}$, $\sigma_{\exp}=725\;\mathrm{MPa}$, the numerical ones are with $E_{macro}=68.6\;\mathrm{GPa}$, $\sigma_{macro}=715.44\;\mathrm{MPa}$). The major fracture initiation and evolution, such as S11 was displayed in Figure 12. The 0 layers undertake the essential loading before their ultimate failure, which determinates its ultimate strengths in integrity as shown in Figure 12c. Once the failure strengths threshold in the 11 direction is satisfied as listed in Table 3, the smear crack with its surface perpendicular to the fiber direction (in Figure 2i) would be initiated numerically and the fiber fractured thoroughly, such as the white area in Figure 12c.

**Figure 13:**
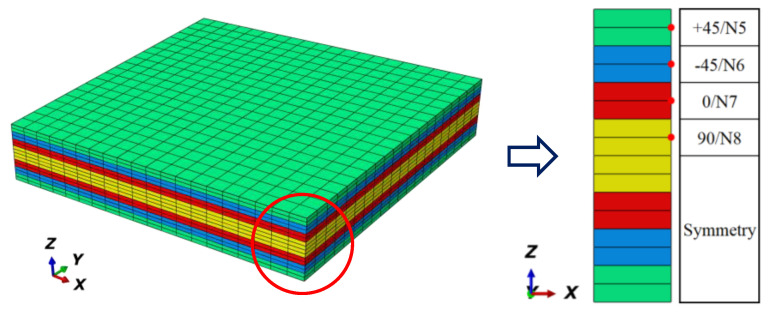
Node selections and their locations of each layer in macro-RVE.

**Figure 14:**
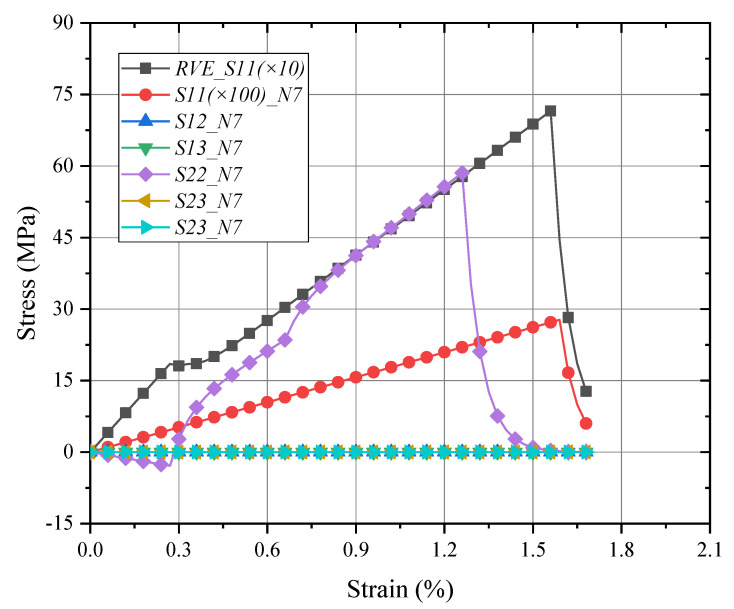
Local responses of 0 layer in macro-RVE under x direction tensile loading.

To reveal the local response of the macro-RVE under the x direction tensile loading, one node of each layer in the middle is selected, respectively, as shown in Figure 13, and its local damage behaviors (such as *N7* for 0° layer) are shown in Figure 14. The stress components, e,g, *S*_11_ and *S*_22_, experienced linear increase, damage initiation/propagation and a sudden degradation. The other stress components undertake low stress levels (far beyond the failure thresholds). The fiber peels damage in the y direction occurred firstly and fiber fractured followed in the x direction. The tensile x direction strengths (*S*_11_) in the 0° degree layer determined the tensile primary integrated strengths (*RVE_S*_11_) of macro-RVE.

### 4.4. Damage Procedure Validations

**Figure 15:**
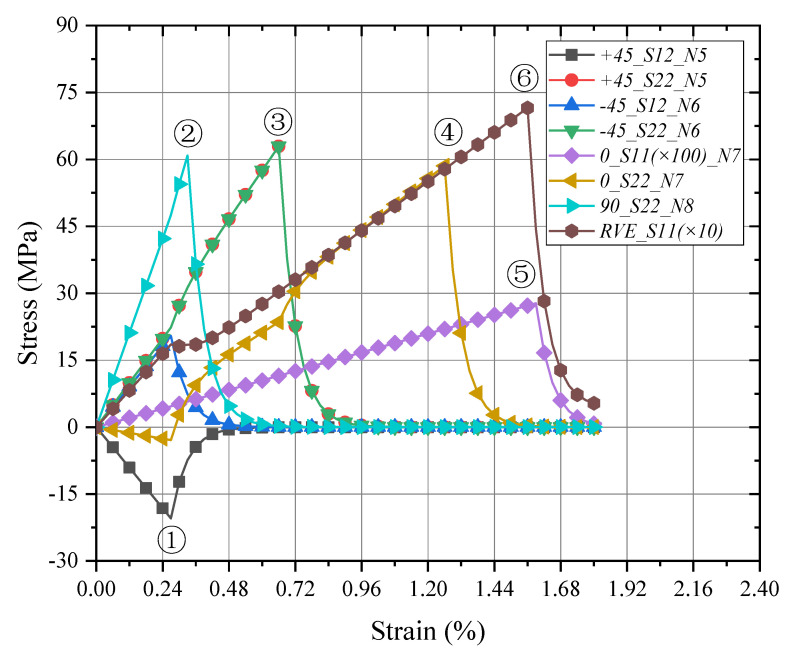
Global/local responses of macro-RVE and layers under x direction tensile loading. Note: ①–⑥ means the stress component peak procedure in different layer.

In the tensile loading process of the macro-RVE, the damage procedure responses in each layer are shown in Figure 15. Compared to the failure strengths as listed in Table 3, the cracks, such as $S_{12}$ (with the peak value of $S_{12}=20.5\;\mathrm{MPa}$) of node 5 and 6 in +45/−45 layers, were firstly initiated at a strain of ε=0.27% (Note: Δε=0.03% for macro-RVE in the x direction tensile loading at each iteration) due to the $\tau_{12}$ shear loading (with failure strength of $\tau_{12}=20.1\;\mathrm{MPa}$), which means the fiber fractured with the effect of transverse shear loading. In the current stage, the global behavior of macro-RVE could be found with a kink corner. Secondly, node 8 displayed the 90 layer to be cracked at a strain of ε=0.33% with $S_{22}$ (with the peak value of $S_{22}=60.9\;\mathrm{MPa}$) is perpendicular to the x direction loading direction due to the transverse tensile loadings ($\sigma_{22t}=57.6\;\mathrm{MPa}$), implying the matrix crack occurred around or among the fibers. Thirdly nodes 5 and 6 in +45/−45 layers are cracked similarly with node 8 in the 90 layer as $S_{22}$ (with the peak value of $S_{22}=63.0\;\mathrm{MPa}$) due to the transverse normal tension at a strain of ε=0.66%. Followingly, the *S*_22_ ($S_{22}=58.6\;\mathrm{MPa}$) in node 7 of the 0° layer is degraded at a strain of ε=1.26% in the resin-rich zone among fiber with the primacy effect of transverse loadings. Upon the further loading at a strain of ε=1.59%, the fiber fractured finally with a peak value of $S_{22}=2774.8\;\mathrm{MPa}$ correspondingly to fiber failure strengths of $\sigma_{11t}=2684.4\;\mathrm{MPa}$, leaving the averaged failure strength of macro-RVE with $S_{11}=714.5\;\mathrm{MPa}$.

**Figure 16:**
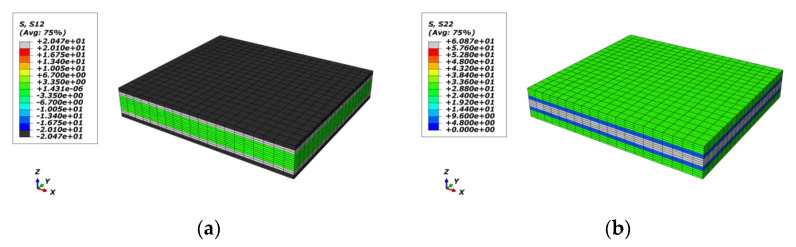

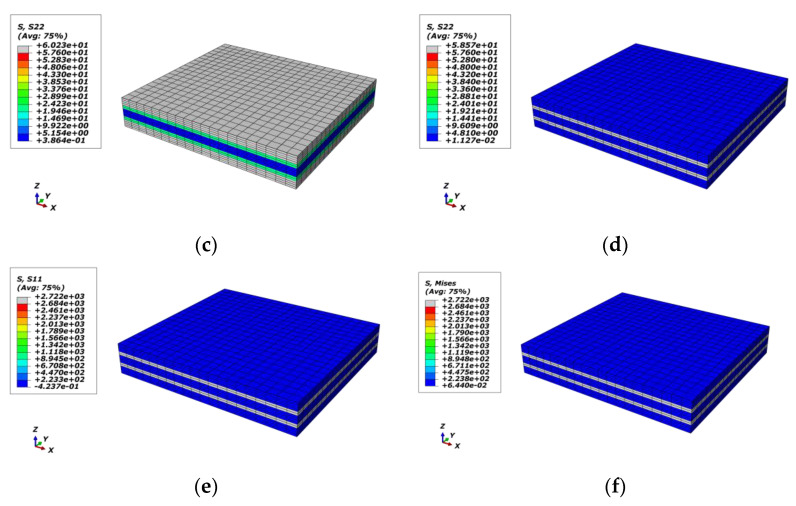
Damage evolution of each layer in macro-RVE under x direction tensile loading: (**a**) $S_{12}/$± 45/${\bar{\varepsilon }}_{11}=0.27\%$; (**b**) $S_{22}/$90/${\bar{\varepsilon }}_{11}=0.33\%$; (**c**) $S_{22}/$± 45/${\bar{\varepsilon }}_{11}=0.66\%$; (**d**) $S_{22}/$0/${\bar{\varepsilon }}_{11}=1.26\%$; (**e**) $S_{11}/$90/${\bar{\varepsilon }}_{11}=1.59\%$; (**f**) S/RVE/${\bar{\varepsilon }}_{11}=1.59\%$.

**Figure 17:**
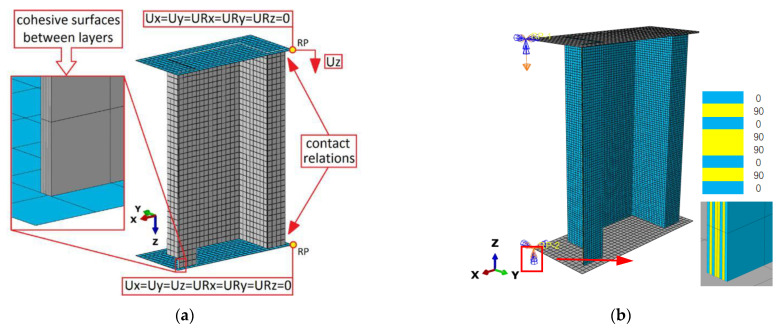
Virtual test of FE models with load and boundary conditions: (**a**) 2D model with shell element (SC8R) and Cohesive element [31]; (**b**) 3D model with solid element (C3D8R).

Corresponding to the above-mentioned cracks in Figure 15, the different damage stages of the macro-RVE are provided in Figure 16, respectively. For each damage stage, the failure strength, as listed in Table 3 (e.g.,$\;S_{12}=20.1\;\mathrm{MPa}$ for red zone in Figure 17a) is set as the stress ultimate threshold of current stress. Once the stress component peak value reaches the failure threshold, the failed elements would be changed into a gloomy or black color. In the loading process, the damage is found firstly from crack initiation from ±45 layers ($S_{12}$) to crack evolution in 90/0 layers ($S_{22}$) and sudden breakage in 0 layers ($S_{11}$). A similar experimental damage mechanism was noticed by previous acoustic emission (AE) and digital image correlation (DIC) findings [36,37,43,44]. At the ply level, the damage evolution typically begins with a “sub-critical” stage of crack formation in the transverse or off-axis plies. The sub-critical damage refers to the formation and multiplication of intralaminar ply cracks, which span through the ply thickness and propagate along the fiber direction, and this process is described as tunneling. Multiplication of ply cracks can occur, due to the constraining effect imposed by the adjacent plies on the cracking plies in the laminate. This sub-critical stage is often followed by the growth of “critical” damage mechanisms, such as delamination, fiber breakage and fiber micro-buckling, which usually leads to a catastrophic laminate failure [45].

## 5. The Extended Validation of UMAT

For further validation of UMATs developed in current multi-scale analysis, failure analysis of thin-walled composite structures using an independent advanced damage model is introduced and compared [31]. Thin-walled composite structures with a top-hat cross-section subjected to axial compression were tested with experimental testing. Both post-critical equilibrium paths and acoustic emission signals were recorded, and the corresponding numerical simulations were realized using progressive fracture analysis (PFA) and the cohesive zone model (CZM) detailed in [31]. According to the FE model (e.g., Figure 17a), the 3D FM model is re-established with the same size, and mechanical properties of the laminar and load/fixed boundary conditions as displayed in Figure 17b.

**Figure 18:**
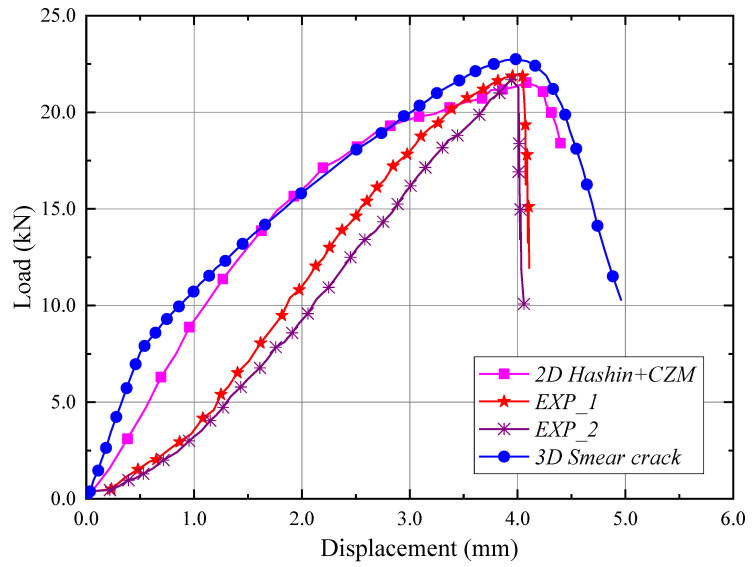
Numerical responses and validations of thin-walled composite structures with 2D model [31] and 3D model.

The results of numerical calculations (numerical model utilizing PFA and CZM analyses) are compared with experimental results from a universal testing machine [31], as shown in Figure 18. Another numerical strategy to feature the global response is used to investigate an in-depth analysis of the previous experimental testing. To develop numerous experimental characteristics, which are compared to the results of damage to the thin-walled composite structure (loss of load-carrying capacity with delamination) obtained using the finite elements method. The performed numerical analyses contributed significantly to the assessment of the complex phenomenon of damage to thin-walled composite structures with top-hat open cross-sections.

Compared to previous experimental and FEA curves, the current numerically obtained curve shows more “stiffness” in the initial range as shown in Figure 18, which is caused by the fact that the numerical models use the assumption of perfect construction, not affected by manufacturing imperfections. In addition, the difference in the initial range of previous and current numerical testing is due to with and without the effect of the interface phase between the 0/90 layers. Nevertheless, a very high agreement of results is obtained, especially in the context of limit loads.

**Figure 19:**
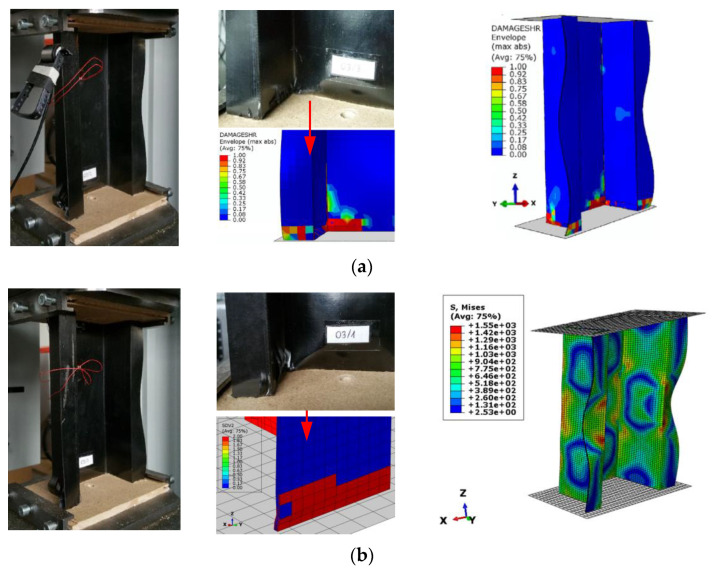
Global deformation and local damage of thin-walled composite structures: (**a**) Experimental and 2D numerical model (PFA and CZM) [31]; (**b**) Experimental [31] and 3D numerical model (On concept of smear crack).

From the macroscopic assessment of the two specimens, as shown in Figure 19, it was proven that damage connected to delamination occurred in the bottom part of the cross-section of the composite column with further validation from previous and current predictions, together with consistent global deformation. Such approaches allowed one to assess the loss of load capacity along with demonstrating the delamination phenomenon.

## 6. Conclusions

The mechanical responses with the failure strengths and damage procedure of angle-ply laminate composite were investigated experimentally and numerically using the multi-RVEs models with self-developed multiple smear crack modes. On the principle of quasi-real micro-structures of fiber packed patterns and the same equivalent fiber volume fraction, the micro-representative volume element model (Micro-RVE) representing the fiber packed zone in the prepreg, and meso-RVE on behalf of a prepreg was constructed. The macro-RVE was established using the homogenization method from different distributions of meso-RVEs. Then multi-scale single/double/multiple smear crack modes were developed and incorporated into user-defined material subroutines (UMATs). Based on the properties of pure fiber and resin, the micro-RVE and meso-RVE’s engineering constants with failure strengths were predicted, respectively. The essential mechanical responses with the damage evolution of APLC were obtained using the macro-RVE analysis. The detailed damage initiation and propagation, including the final damage morphologies, were featured in detail. The numerical tensile modulus, failure strengths and damage procedure were compared to experimental results. Upon further loading processes, the transverse cracks in the 90/±45 layer, such as S22/S12 are initiated at the primary stage. The 0 fiber layer determines the ultimate failure strengths of macro-RVE.

This micro/meso/macroscale analysis with user-defined UMATs can provide a strategy to investigate the global/local responses and damage procedures of composite structures under the uniaxial or even multi-axial loadings.

## Figures and Tables

**Table 1:** Fiber and resin properties in T800 carbon fiber prepreg.

Property	Value
$\mathrm{Young}’s\;\mathrm{modulus}\;\mathrm{of}\;\mathrm{fiber}:\;E_{f}$ (GPa)	300
$\mathrm{Poisson}’s\;\mathrm{ratio}\;\mathrm{of}\;\mathrm{fiber}:\;\mu_{f}$	0.22
$\mathrm{Tensile}\;\mathrm{strengths}\;\mathrm{of}\;\mathrm{fiber}:\;\sigma_{tf}$ (GPa)	4.5
$\mathrm{Compressive}\;\mathrm{strengths}\;\mathrm{of}\;\mathrm{fiber}:\;\sigma_{cf}$ (GPa)	−2.25
$\mathrm{Shear}\;\mathrm{strengths}\;\mathrm{of}\;\mathrm{fiber}:\;\sigma_{sf}$ (GPa)	1.23
$\mathrm{Tensile}\;\mathrm{modulus}\;\mathrm{of}\;\mathrm{matrix}:\;E_{m}$ (GPa)	3.6
$\mathrm{Poisson}’s\;\mathrm{ratio}\;\mathrm{of}\;\mathrm{matrix}:\;\mu_{m}$	0.4
$\mathrm{Tensile}\;\mathrm{strengths}\;\mathrm{of}\;\mathrm{matrix}:\;\sigma_{tm}$ (GPa)	72
$\mathrm{Compressive}\;\mathrm{strengths}\;\mathrm{of}\;\mathrm{matrix}:\;\sigma_{cm}$ (MPa)	−36
$\mathrm{Shear}\;\mathrm{strengths}\;\mathrm{of}\;\mathrm{fiber}:\;\sigma_{sm}$ (MPa)	25.7

**Table 2:** Mechanical parameters of micro-RVE.

Property	Value
$\mathrm{Longitudinal}\;\mathrm{Young}’s\;\mathrm{modulus}\;\mathrm{of}\;\mathrm{fiber}:\;E_{11}$ (GPa)	206.00
$\mathrm{Transvers}\;\mathrm{Young}’s\;\mathrm{modulus}:\;\;E_{22}$ (GPa)	22.20
$\mathrm{Transvers}\;\mathrm{Young}’s\;\mathrm{modulus}:\;E_{33}$ (GPa)	22.10
$\mathrm{Shear}\;\mathrm{modulus}:\;G_{12}$ (GPa)	6.60
$\mathrm{Shear}\;\mathrm{modulus}:\;G_{13}$ (GPa)	6.60
$\mathrm{Shear}\;\mathrm{modulus}:\;G_{23}$ (GPa)	7.60
$\mathrm{Poisson}’s\;\mathrm{ratio}:\;\mu_{12}$	0.27
$\mathrm{Poisson}’s\;\mathrm{ratio}:\;\mu_{13}$	0.27
$\mathrm{Poisson}’s\;\mathrm{ratio}:\;\mu_{23}$	0.47
$\mathrm{Longitudinal}\;\mathrm{tensile}\;\mathrm{strength}:\;\sigma_{11}^{t}$ (MPa)	3131.50
$\mathrm{Longitudinal}\;\mathrm{compressive}\;\mathrm{strength}:\;\sigma_{11}^{c}$ (MPa)	−1568.80
$\mathrm{Transverse}\;\mathrm{tensile}\;\mathrm{strength}:\;\sigma_{22}^{t}$ (MPa)	63.60
$\mathrm{Transverse}\;\mathrm{compressive}\;\mathrm{strength}:\;\sigma_{22}^{c}$ (MPa)	−40.70
$\mathrm{Transverse}\;\mathrm{tensile}\;\mathrm{strength}:\;\sigma_{33}^{t}$ (MPa)	63.80
$\mathrm{Transverse}\;\mathrm{compressive}\;\mathrm{strength}:\;\sigma_{33}^{c}$ (MPa)	−33.30
$\mathrm{Shear}\;\mathrm{strength}:\;\tau_{12}$ (MPa)	22.70
$\mathrm{Shear}\;\mathrm{strength}:\;\tau_{13}$ (MPa)	20.50
$\mathrm{Shear}\;\mathrm{strength}:\;\tau_{23}$ (MPa)	24.10

**Table 3:** Mechanical parameters of meso-RVE.

Property	Value
$\mathrm{Longitudinal}\;\mathrm{Young}’s\;\mathrm{modulus}:\;E_{11}$ (GPa)	173.20
$\mathrm{Transvers}\;\mathrm{Young}’s\;\mathrm{modulus}:\;E_{22}$ (GPa)	19.20
$\mathrm{Transvers}\;\mathrm{Young}’s\;\mathrm{modulus}:\;E_{33}$ (GPa)	15.60
$\mathrm{In}-\mathrm{plane}\;\mathrm{shear}\;\mathrm{modulus}:\;G_{12}$ (GPa)	5.70
$\mathrm{Out}-\mathrm{plane}\;\mathrm{shear}\;\mathrm{modulus}:\;G_{13}$ (GPa)	4.00
$\mathrm{Out}-\mathrm{plane}\;\mathrm{shear}\;\mathrm{modulus}:\;G_{23}$ (GPa)	4.20
$\mathrm{In}-\mathrm{plane}\;\mathrm{Poisson}’s\;\mathrm{ratio}:\;\mu_{12}$	0.2747
$\mathrm{Out}-\mathrm{plane}\;\mathrm{Poisson}’s\;\mathrm{ratio}:\;\mu_{13}$	0.3027
$\mathrm{Out}-\mathrm{plane}\;\mathrm{Poisson}’s\;\mathrm{ratio}:\;\mu_{23}$	0.4981
$\mathrm{Longitudinal}\;\mathrm{tensile}\;\mathrm{strength}:\;\sigma_{11}^{t}$ (MPa)	2684.4
$\mathrm{Longitudinal}\;\mathrm{compressive}\;\mathrm{strength}:\;\sigma_{11}^{c}$ (MPa)	−1385.5
$\mathrm{Intralaminar}\;\mathrm{normal}\;\mathrm{tensile}\;\mathrm{strength}:\;\sigma_{22}^{t}$ (MPa)	57.60
$\mathrm{Intralaminar}\;\mathrm{normal}\;\mathrm{compressive}\;\mathrm{strength}:\;\sigma_{22}^{c}$ (MPa)	−38.40
$\mathrm{Interfacial}\;\mathrm{normal}\;\mathrm{tensile}\;\mathrm{strength}:\;\sigma_{33}^{t}$ (MPa)	70.40
$\mathrm{Interfacial}\;\mathrm{normal}\;\mathrm{compressive}\;\mathrm{strength}:\;\sigma_{33}^{c}$ (MPa)	−39.10
$\mathrm{Intralaminar}\;\mathrm{shear}\;\mathrm{strength}:\;\tau_{12}$ (MPa)	20.10
$\mathrm{Interfacial}\;\mathrm{Shear}\;\mathrm{strength}:\;\tau_{13}$ (MPa)	21.80
$\mathrm{Interfacial}\;\mathrm{Shear}\;\mathrm{strength}:\;\tau_{23}$ (MPa)	25.40

**Table 4:** Mechanical parameters of macro-RVE.

Property	Value
$x\;\mathrm{direction}’s\;\mathrm{Young}’s\;\mathrm{modulus}:\;E_{11}$ (GPa)	68.60
$y\;\mathrm{direction}’s\;\mathrm{Young}’s\;\mathrm{modulus}:\;\;E_{22}$ (GPa)	68.60
$z\;\mathrm{direction}’s\;\mathrm{Young}’s\;\mathrm{modulus}:\;E_{33}$ (GPa)	18.30
$\mathrm{In}-\mathrm{plane}\;\mathrm{shear}\;\mathrm{modulus}:\;G_{12}$ (GPa)	25.8
$\mathrm{Out}-\mathrm{plane}\;\mathrm{shear}\;\mathrm{modulus}:\;G_{13}$ (GPa)	4.10
$\mathrm{Out}-\mathrm{plane}\;\mathrm{shear}\;\mathrm{modulus}:\;G_{23}$ (GPa)	4.10
$\mathrm{In}-\mathrm{plane}\;\mathrm{Poisson}’s\;\mathrm{ratio}:\;\mu_{12}$	0.3303
$\mathrm{Out}-\mathrm{plane}\;\mathrm{Poisson}’s\;\mathrm{ratio}:\;\mu_{13}$	0.3199
$\mathrm{Out}-\mathrm{plane}\;\mathrm{Poisson}’s\;\mathrm{ratio}:\;\mu_{23}$	0.3199
$x\;\mathrm{direction}’s\;\mathrm{tensile}\;\mathrm{strength}:\;\sigma_{11}^{t}$ (MPa)	715.44
$x\;\mathrm{direction}’s\;\mathrm{compressive}\;\mathrm{strength}:\;\sigma_{11}^{c}$ (MPa)	−317.97
$y\;\mathrm{direction}’s\;\mathrm{tensile}\;\mathrm{strength}:\;\sigma_{22}^{t}$ (MPa)	715.44
$y\;\mathrm{direction}’s\;\mathrm{compressive}\;\mathrm{strength}:\;\sigma_{22}^{c}$ (MPa)	−317.97
$z\;\mathrm{direction}’s\;\mathrm{tensile}\;\mathrm{strength}:\;\sigma_{33}^{t}$ (MPa)	71.5
$z\;\mathrm{direction}’s\;\mathrm{compressive}\;\mathrm{strength}:\;\sigma_{33}^{c}$ (MPa)	−44.00
$\mathrm{In}-\mathrm{plane}\;\mathrm{shear}\;\mathrm{strength}:\;\tau_{12}$ (MPa)	355.47
$\mathrm{Out}-\mathrm{plane}\;\mathrm{shear}\;\mathrm{strength}:\;\tau_{13}$ (MPa)	22.10
$\mathrm{Out}-\mathrm{plane}\;\mathrm{shear}\;\mathrm{strength}:\;\tau_{23}$ (MPa)	22.10

## Data Availability

All data included in this study are available upon reasonable request by contact with the corresponding author.

## References

[1] Pinho S., Robinson P., Iannucci L. (2006). Fracture toughness of the tensile and compressive fibre failure modes in laminated composites. Compos. Sci. Technol..

[2] Wang X., Guan Z., Du S., Han G., Li Z. (2020). An accurate and easy to implement method for predicting matrix crack and plasticity of composites with an efficient search algorithm for LaRC05 criterion. Compos. Part A Appl. Sci. Manuf..

[3] Talreja R. (2014). Assessment of the fundamentals of failure theories for composite materials. Compos. Sci. Technol..

[4] Fakoor M., Shahsavar S. (2020). Fracture assessment of cracked composite materials: Progress in models and criteria. Theor. Appl. Fract. Mech..

[5] Parambil N.K., Gururaja S. (2019). Bridging micro-to-macro scale damage in UD-FRP laminates under tensile loading. Int. J. Mech. Sci..

[6] Zhuang L., Talreja R., Varna J. (2018). Transverse crack formation in unidirectional composites by linking of fibre/matrix debond cracks. Compos. Part A Appl. Sci. Manuf..

[7] Maragoni L., Talreja R. (2019). Transverse crack formation in unidirectional plies predicted by means of a percolation concept. Compos. Part A Appl. Sci. Manuf..

[8] Huang Y., Varna J., Talreja R. (2014). Statistical methodology for assessing manufacturing quality related to transverse cracking in cross ply laminates. Compos. Sci. Technol..

[9] Melro A., Camanho P., Pires F., Pinho S. (2013). Micromechanical analysis of polymer composites reinforced by unidirectional fibres: Part I–Constitutive modelling. Int. J. Solids Struct..

[10] Melro A., Camanho P., Pires F., Pinho S. (2013). Micromechanical analysis of polymer composites reinforced by unidirectional fibres: Part II–Micromechanical analyses. Int. J. Solids Struct..

[11] Naderi M., Iyyer N. (2020). Micromechanical analysis of damage mechanisms under tension of 0°–90° thin-ply composite laminates. Compos. Struct..

[12] Elnekhaily S.A., Talreja R. (2019). Effect of axial shear and transverse tension on early failure events in unidirectional polymer matrix composites. Compos. Part A Appl. Sci. Manuf..

[13] Bullegas G., Lamela J.M., Pimenta S., Pinho S.T. (2020). On the role of dynamic stress concentrations and fracture mechanics in the longitudinal tensile failure of fibre-reinforced composites. Eng. Fract. Mech..

[14] Chen B., Tay T., Pinho S., Tan V. (2017). Modelling delamination migration in angle-ply laminates. Compos. Sci. Technol..

[15] Pimenta S., Gutkin R., Pinho S., Robinson P. (2009). A micromechanical model for kink-band formation: Part I—Experimental study and numerical modelling. Compos. Sci. Technol..

[16] Pimenta S., Gutkin R., Pinho S., Robinson P. (2009). A micromechanical model for kink-band formation: Part II—Analytical modelling. Compos. Sci. Technol..

[17] Andraju L.B., Raju G. (2020). Continuum and cohesive zone damage models to study intra/inter-laminar failure of curved composite laminates under four-point bending. Compos. Struct..

[18] Forghani A., Poursartip A., Vaziri R. (2019). An orthotropic non-local approach to modeling intra-laminar damage progression in laminated composites. Int. J. Solids Struct..

[19] Boon Y.D., Joshi S.C. (2020). A review of methods for improving interlaminar interfaces and fracture toughness of laminated composites. Mater. Today Commun..

[20] Farrokhabadi A., Babaei R. (2019). Development of an integrated micro macro model for anticipating matrix cracking evolution and fiber breakage in the laminated composite containing an open hole. Eng. Fract. Mech..

[21] Ren M.-F., Zhang X.-W., Huang C., Wang B., Li T. (2019). An integrated macro/micro-scale approach for in situ evaluation of matrix cracking in the polymer matrix of cryogenic composite tanks. Compos. Struct..

[22] Patel D.K., Waas A.M. (2019). Multiscale analysis of notched fiber reinforced laminates. Compos. Part B Eng..

[23] Massarwa E., Aboudi J., Haj-Ali R. (2019). A multiscale modeling for failure predictions of fiber reinforced composite laminates. Compos. Part B Eng..

[24] Yuan M., Yang Y., Zhao H., Wang Y., Li R., Zhang B., Chen J. (2020). A novel trans-scale method for predicting mode I matrix crack density of composite laminates. Compos. Struct..

[25] Laux T., Gan K.W., Dulieu-Barton J., Thomsen O.T. (2020). Ply thickness and fibre orientation effects in multidirectional composite laminates subjected to combined tension/compression and shear. Compos. Part A Appl. Sci. Manuf..

[26] Kumagai Y., Onodera S., Salviato M., Okabe T. (2020). Multiscale analysis and experimental validation of crack initiation in quasi-isotropic laminates. Int. J. Solids Struct..

[27] Jia X., Xia Z., Gu B. (2013). Numerical analyses of 3D orthogonal woven composite under three-point bending from multi-scale microstructure approach. Comput. Mater. Sci..

[28] Jia X., Xia Z., Gu B. (2013). Nonlinear viscoelastic multi-scale repetitive unit cell model of 3D woven composites with damage evolution. Int. J. Solids Struct..

[29] Tan P. (2014). Numerical simulation of the ballistic protection performance of a laminated armor system with pre-existing debonding/delamination. Compos. Part B Eng..

[30] Kolanu N.R., Raju G., Ramji M. (2020). A unified numerical approach for the simulation of intra and inter laminar damage evolution in stiffened CFRP panels under compression. Compos. Part B Eng..

[31] Rozylo P. (2021). Failure analysis of thin-walled composite structures using independent advanced damage models. Compos. Struct..

[32] Almeida J.H.S., Ribeiro M.L., Tita V., Amico S.C. (2016). Damage and failure in carbon/epoxy filament wound composite tubes under external pressure: Experimental and numerical approaches. Mater. Des..

[33] Almeida J.H.S., Ribeiro M., Tita V., Amico S. (2017). Damage modeling for carbon fiber/epoxy filament wound composite tubes under radial compression. Compos. Struct..

[34] Almeida J.H.S., Tonatto M., Ribeiro M.L., Tita V., Amico S. (2018). Buckling and post-buckling of filament wound composite tubes under axial compression: Linear, nonlinear, damage and experimental analyses. Compos. Part B Eng..

[35] Almeida J.H.S., St-Pierre L., Wang Z., Ribeiro M.L., Tita V., Amico S.C., Castro S.G. (2021). Design, modeling, optimization, manufacturing and testing of variable-angle filament-wound cylinders. Compos. Part B Eng..

[36] Oz F.E., Ersoy N., Mehdikhani M., Lomov S.V. (2018). Multi-instrument in-situ damage monitoring in quasi-isotropic CFRP laminates under tension. Compos. Struct..

[37] Li D., Guo Q., Xu D., Yang X. (2017). Three-dimensional micromechanical analysis models of fiber reinforced composite plates with damage. Comput. Struct..

[38] Jia X., Xia Z., Gu B. (2012). Micro/meso-scale damage analysis of three-dimensional orthogonal woven composites based on sub-repeating unit cells. J. Strain Anal. Eng. Des..

[39] Malvar L., Fourney M. (1990). A three dimensional application of the smeared crack approach. Eng. Fract. Mech..

[40] De Borst R. (1987). Smeared cracking, plasticity, creep, and thermal loading—A unified approach. Comput. Methods Appl. Mech. Eng..

[41] Zhang Y., Xia Z., Ellyin F. (2005). Nonlinear viscoelastic micromechanical analysis of fibre-reinforced polymer laminates with damage evolution. Int. J. Solids Struct..

[42] Ellyin F., Zhang Y., Xia Z. (2011). Meso-scale analysis of angle-ply laminates. Procedia Eng..

[43] Han W., Hu K., Shi Q., Zhu F. (2020). Damage evolution analysis of open-hole tensile laminated composites using a progress damage model verified by AE and DIC. Compos. Struct..

[44] Oz F.E., Mehdikhani M., Ersoy N., Lomov S.V. (2020). In-situ imaging of inter- and intra-laminar damage in open-hole tension tests of carbon fibre-reinforced composites. Compos. Struct..

[45] Sharifpour F., Montesano J., Talreja R. (2020). Assessing the effects of ply constraints on local stress states in cross-ply laminates containing manufacturing induced defects. Compos. Part B Eng..

